# Supplementary material for: Humidity-tolerant rate-dependent capillary viscous adhesion of bee-collected pollen fluids
Source: Nat Commun. 2019 Mar 26;10:1379. doi: 10.1038/s41467-019-09372-x (PMC6435648; doi:10.1038/s41467-019-09372-x)
Supplement: Supplementary file 1 — Supplementary Information [file 41467_2019_9372_MOESM1_ESM.pdf]

Supplementary Information

Humidity-Tolerant Rate-Dependent Capillary Viscous Adhesion of Bee-Collected Pollen Fluids

Shin et al.

Supplementary Information

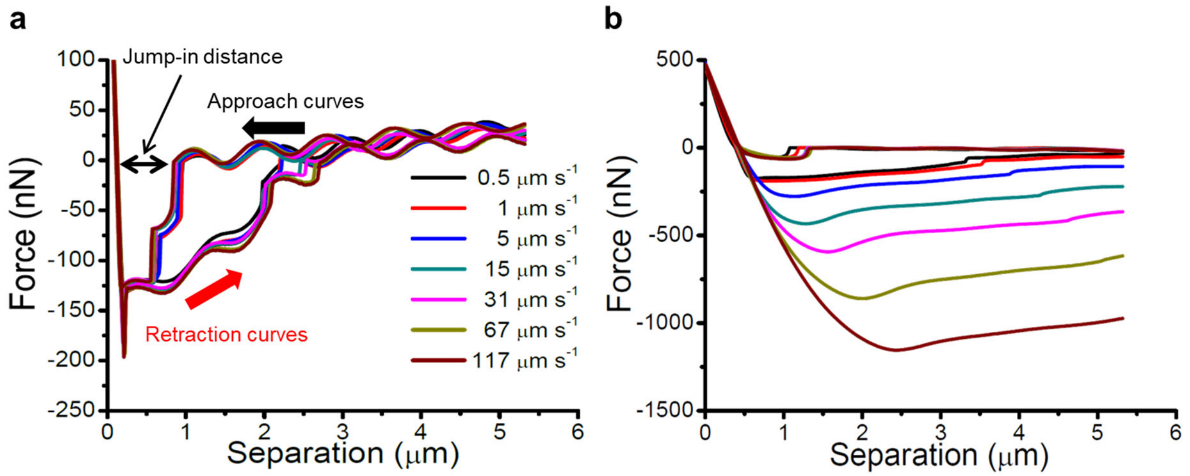

Supplementary Figure 1 | AFM force-distance curves at different separation rates from 0.5 μm s^{-1} to 117 μm s^{-1} Adhesion of a cleaned sunflower pollen (*Helianthus annuus*) on (**a**) surrounding and (**b**) core regions of a bee pollen adhesive droplet. The first minimum in the retraction curves corresponds to the maximum capillary adhesive achieved. The multiple step-like features on the retraction curves are attributed to the multiple liquid bridges on individual spines.

Supplementary Table 1 | Physical properties of the aqueous and the oily phases (extracted and separated using solvent extraction as described in Methods) in bee pollen adhesive

	Solvent	Mass fraction	Density (g ml^{-1})	Surface tension (mN m^{-1})
Aqueous phase	Water	0.906	1.547	52.5
Oily phase	Toluene	0.094	0.948	22.5

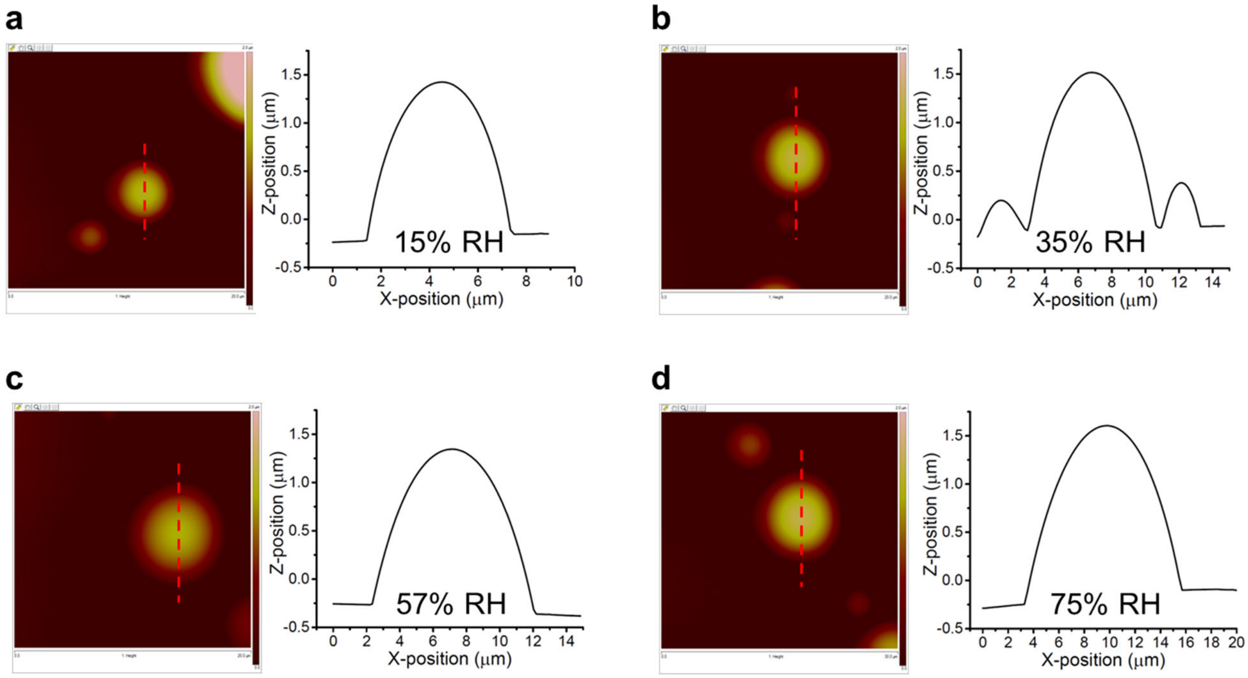

Supplementary Figure 2 | AFM images of the solvent washed bee pollen adhesive droplets on a silicon wafer The droplets were stored in (**a**) 15%, (**b**) 35%, (**c**) 57%, and (**d**) 75% RH for 24h. The x-and z-position graphs represent the cross-sections of the red dashed lines on the droplet images.

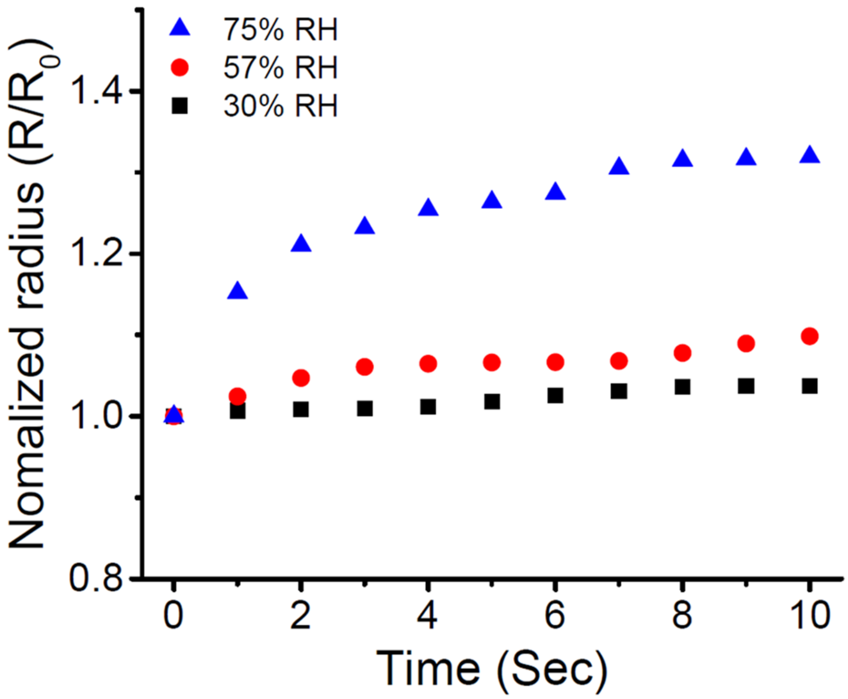

Supplementary Figure 3 | Normalized spreading radius of the aqueous extract droplets (13 ± 2 μL) on a silicon wafer Before spreading the droplets on the surface, 1 ml of three aqueous extract samples was stored in three different humidity levels (30%, 57%, 75% RH) for 3 days. The spreading radius of the droplets was measured at 20°C and at 45% RH. R_0 is the initial radius of the droplet.

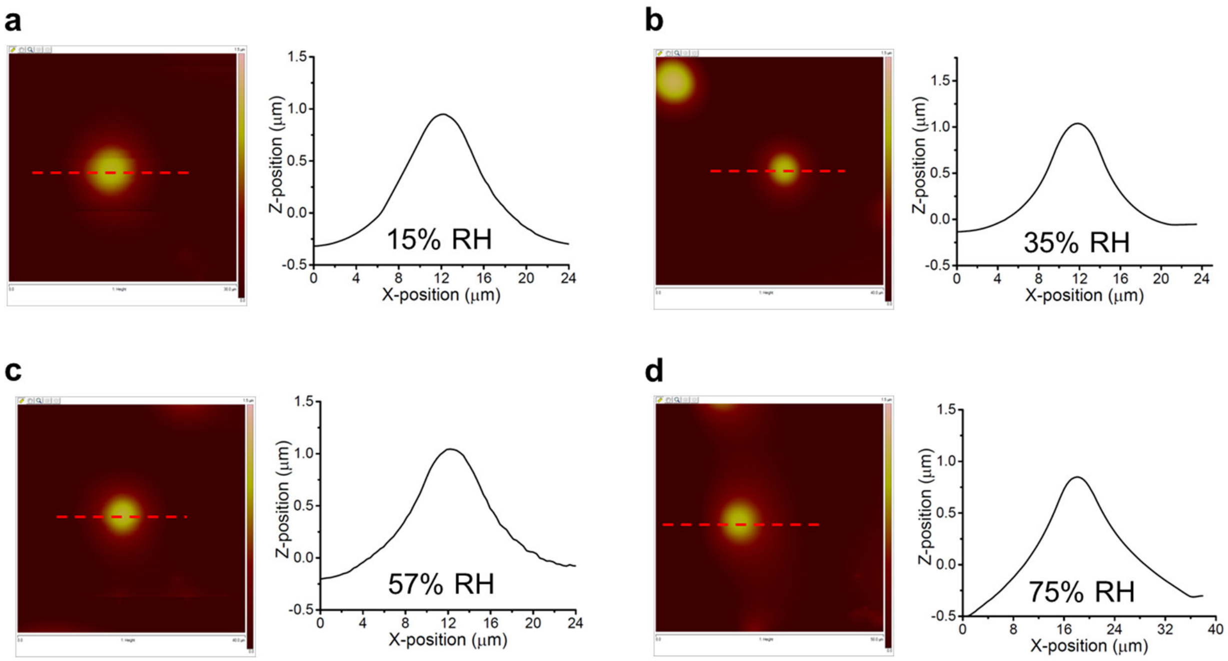

Supplementary Figure 4 │ AFM images of the bee pollen adhesive droplets on a silicon wafer The droplets were stored in (**a**) 15%, (**b**) 35%, (**c**) 57%, and (**d**) 75% RH for 24h. The x-and z-position graphs represent the cross-sections of the red dashed lines on the droplet images.

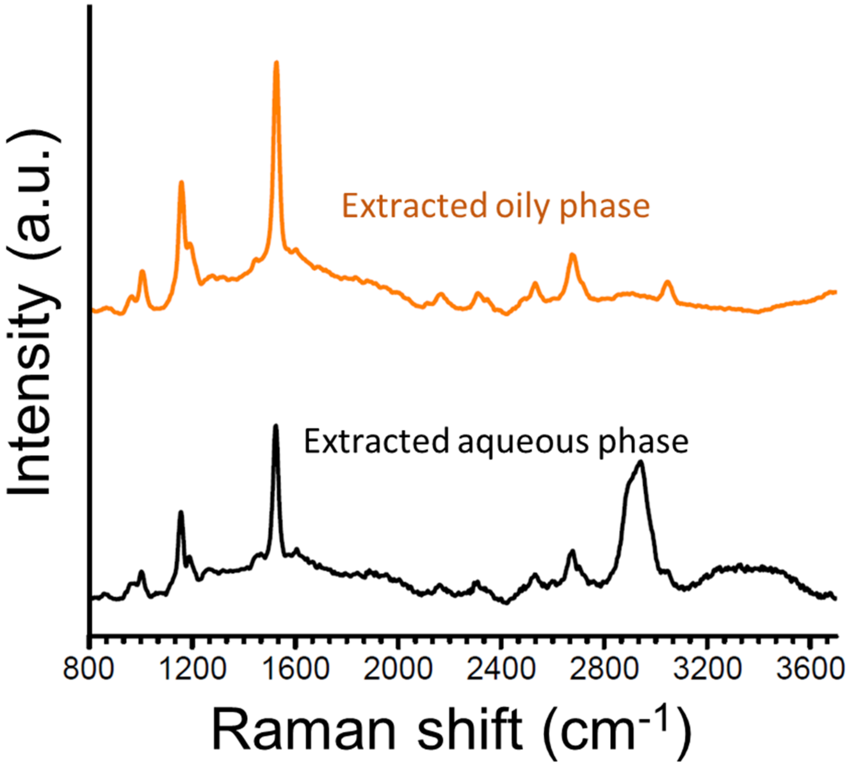

Supplementary Figure 5 | Confocal Raman spectra of extracted phases deposited on Si wafer

The black line and orange lines represent the aqueous and toluene extracts respectively.

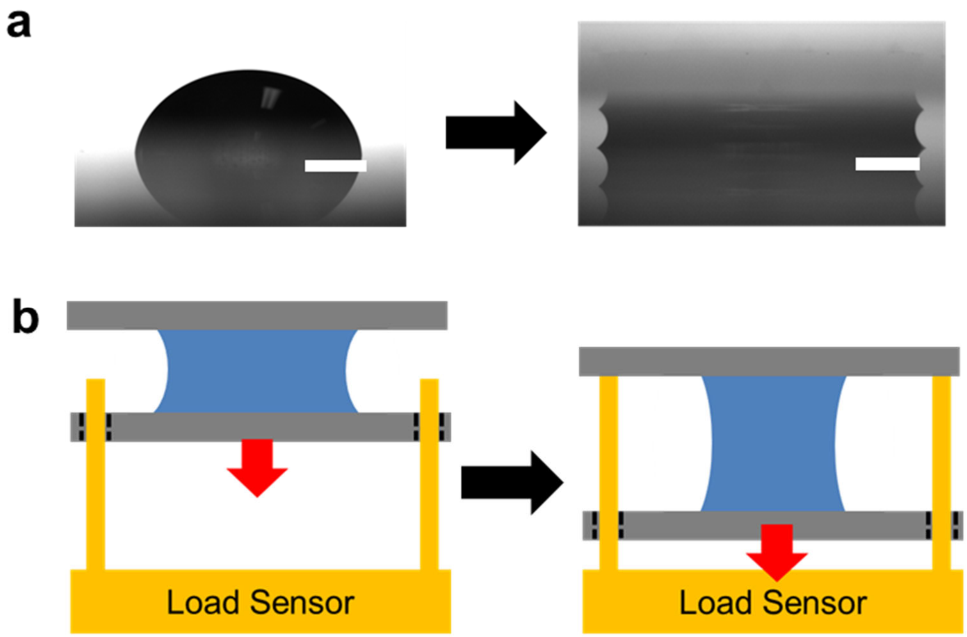

Supplementary Figure 6 | Adhesion force due to the aqueous extract liquid bridge between two glass slides (**a**) Image of the aqueous extract liquid bridge between two glass slides. Scale bar = 1 mm. (**b**) Schematic illustration of the wet adhesion measurement.

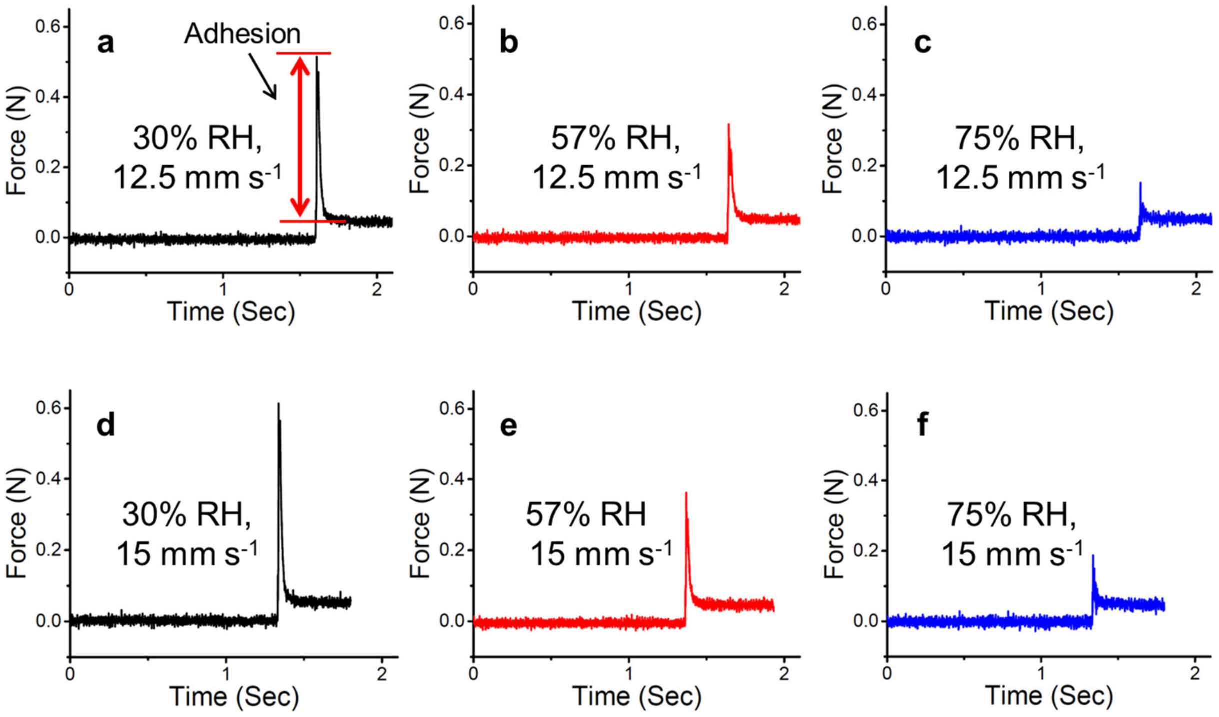

Supplementary Figure 7│ Force versus time measured for the liquid bridges of aqueous extract between two glass plates Separation rates (**a**, **b**, **c**) at 12.5 mm s^{-1} and (**d**, **e**, **f**) at 15 mm s^{-1}. Relative humidity levels (**a**, **d**) at 30% RH, (**b**, **e**) at 57% RH, and (**c**, **f**) at 75% RH After the liquid bridges are broken (at the terminal period), all curves are containing residual forces (46.7 ± 0.5 mN) due to the mass of the upper glass slide (about 4.8 g).

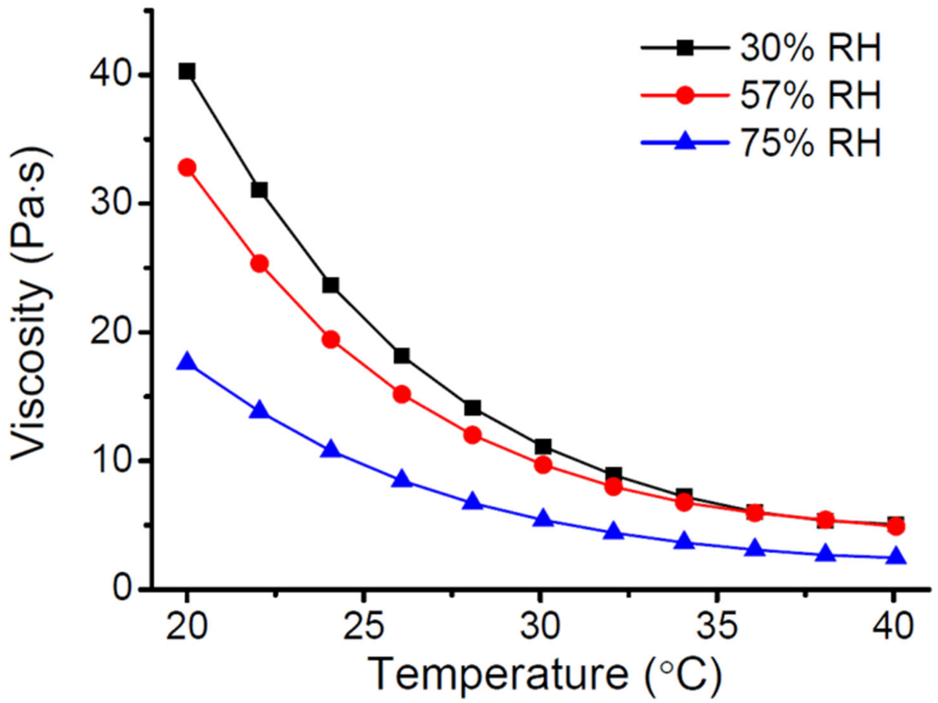

Supplementary Figure 8│ Temperature-dependence of viscosity of the aqueous extract samples The viscosity of the aqueous phase samples stored at different relative humidity levels (30%, 57%, and 75% RH for 3 days) as a function of temperature from 20 to 40 °C.
